# THE ROLE OF POLYGENIC SCORE AND COGNITIVE ACTIVITIES ON COGNITIVE FUNCTIONING OF OLDER ADULTS

**DOI:** 10.1093/geroni/igz038.954

**Published:** 2019-11-08

**Authors:** Su Hyun Shin, Soohyun Park, Giyeon Kim

**Affiliations:** 1 The University of Alabama, Tuscaloosa, Alabama, United States; 2 Chung-Ang University, Seoul, Korea, Republic of

## Abstract

Purpose of study: This study investigated whether and to what extent genetics for cognition and engagement in cognitive activities are related to trajectories of cognitive functioning in older adulthood. Furthermore, we explored whether engaging in cognitive activities could moderate the effect of genetic traits on cognitive functioning in general and across different dimensions: fluid and crystallized intelligence. Design and Methods: Growth curve models were estimated using the sample of 3,129 individuals aged 50 or older (10,000 observations) in the U.S. from 2000-2012 waves of the Health and Retirement Study. Polygenic score for general cognition (PGS) was used to measure genetic traits for cognition, and the number of hours spent per week on each of nine cognitive activities was used to measure individuals’ level of the engagement in cognitive activities. Results: PGS for cognition, reading books, using a computer, and playing cards/games/solving puzzles had positive effects on cognitive functioning. The positive effect of PGS on cognitive functioning was reduced from excessive TV watching. The positive effect of PGS on cognitive functioning was strengthened by spending more hours reading papers/magazines. The measure of fluid, rather than crystallized intelligence, appeared to drive these results. Conclusion: Findings suggests that while genetic factors predict cognitive functioning, engaging in different types of cognitive activities could yield different cognitive functioning trajectories in later life. Practical implications are that older adults should be more selective when choosing their leisure activities to promote cognitive health.

